# Supplementary material for: Directed evolution of the PcaV allosteric transcription factor to generate a biosensor for aromatic aldehydes
Source: J Biol Eng. 2019 Nov 27;13:91. doi: 10.1186/s13036-019-0214-z (PMC6882365; doi:10.1186/s13036-019-0214-z)
Supplement: Supplementary file 1 — Additional file 1: Contains supplementary Figure S1-S6 and Table S1-10. [file 13036_2019_214_MOESM1_ESM.docx]

Additional file

Directed evolution of the PcaV allosteric transcription factor to generate a biosensor for aromatic aldehydes.

Leopoldo F. M. Machado^12^, Andrew Currin^123^, Neil Dixon^123^.

^1^Manchester Institute of Biotechnology (MIB), ^2^Department of Chemistry, ^3^SYNBIOCHEM,

The University of Manchester, Manchester, M1 7DN, UK.

[neil.dixon@manchester.ac.uk](mailto:neil.dixon@manchester.ac.uk)

**Figure S1:** PCA Biosensor promoter-operator construction and substrate specificity screening**. A.** The palindromic DNA operator region O_I_ in *Streptomyces coelicolor* is shown. PcaV binds to the O_I_ sequence regulating the *pcaI* gene as described by Davis et al, 2013 [1]. Two chimeric promoters based in the phage lambda promoter (P_LV_) and the phage T7A1 promoter (P_PV_) were designed using the O_I_ region. **B.** The P_PV_ version of the PCA Biosensor was induced with 29 chemicals at 1 mM concentration. Over a range of aromatic acids the biosensor was specific to PCA (3,4-dyhydroxybenzoic acid), 3-hydroxybenzoic acid and 4-hydroxybenzoic acid.

**
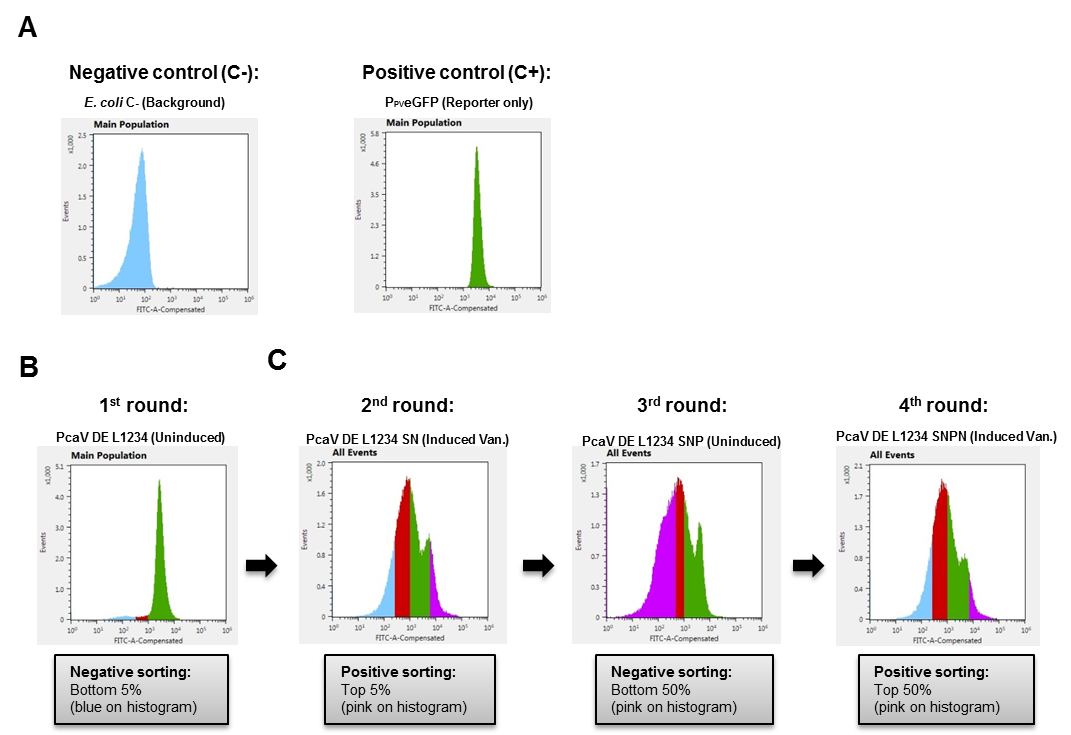
**

**Figure S2:** FACS counter-selection of the L1234 combined library. **A.** Cultures of *E. coli BW25113* (K strain) without plasmids (C-) or transformed with plasmids for the GFP reporter system (C+) were used to set the GFP negative and GFP positive gates respectively. **B.** Rounds of FACS counter-screening were performed alternating negative sorting (library culture on the absence of effector and sorting of the events in the bottom of the fluorescence histogram) and positive sorting (library culture on the presence of the desired effector and sorting of the events in the top of the histogram). In the 1^st^ round, the L1234 library in the absence of effector was used to sort the bottom 5% (blue portion of the histogram). In the 2^nd^ round, the previously sorted library was induced with the effector vanillin at 1 mM followed by sorting of the top 5% (pink portion of the histogram). In the 3^rd^ round, the sorted library culture on absence of effector was used to sort the bottom 50% (pink portion on histogram). In the 4^th^ and final round, the sorted library was induced with the effector vanillin at 1 mM followed by sorting of the top 5% (pink portion of the histogram). This last sorted library was collected for a subsequent plate screening.

**
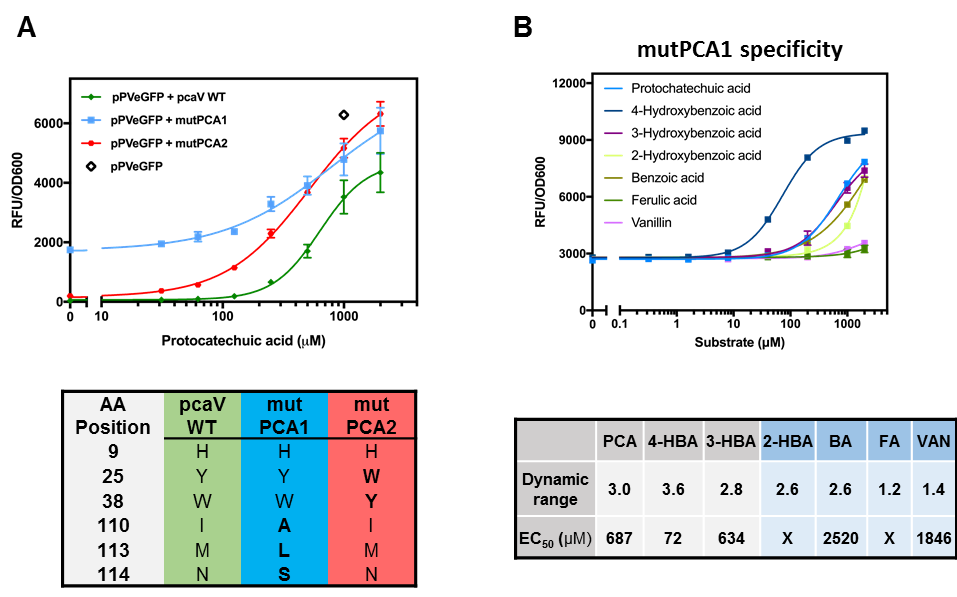
**

**Figure S3:** Two mutants were selected from the PcaV evolution libraries induced with the parental inducer protocatechuic acid. **A.** The mutPCA1 and mutPCA2 amino acids positions on the effector binding-pocket are shown compared to the wild type PcaV. The PcaV WT (green diamonds), mutPCA1 (blue squares) and mutPCA2 (red circles) were induced with increasing concentrations of PCA. The reporter expression is also shown (empty black diamond). **B.** The mutPCA1 induction titration with increasing concentrations of the active substrates was made, showing detection of four additional compounds compared to the PCA Biosensor WT. The dynamic range and EC_50_ are shown on the table. The fluorescent gene expression normalised to cell density (RFU/OD_600_) was plotted, and the dose response curves were fitted. Each value represents the mean and standard deviation of biological triplicates.


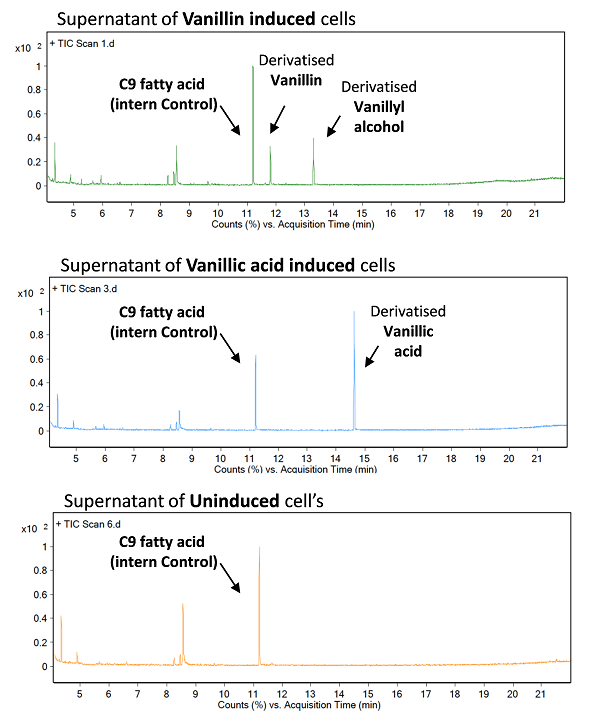


**A**


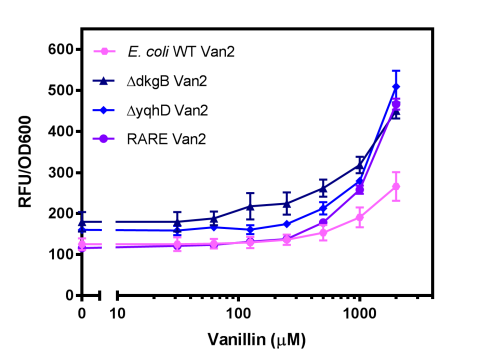


**B**

**C**


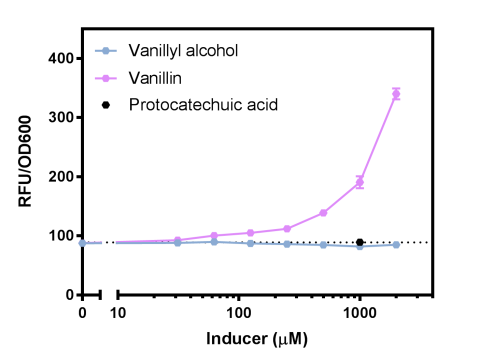


**Figure S4:** The supernatants of *E. coli* BW25113 Van2 Biosensor cells uninduced or induced with vanillin or vanillic acid were analysed by GC-MS to confirm the detection of vanillin. **A.** GC traces of the derivatised supernatants from cells induced with vanillin (green), vanillic acid (blue) and uninduced cells (yellow) are shown. All the supernatants were supplemented with a C9 fatty acid as a GC-MS intern control. Two peaks referring to derivatised vanillin (~11.8 minutes) and derivatised vanillyl alcohol (~13.5 minutes) are observed for vanillin induced cells. One peak referring to derivatised vanillic acid (~14.8 minutes) is only observed for vanillic acid induced cells. The control C9 fatty acid peak is observed (~ 11 min) for all samples. No additional peak is observed for the supernatant of uninduced cells. **B.** The Van2 Biosensor was induced with increasing concentrations of vanillin and vanillyl alcohol and 1 mM of PCA. **C.** KO strains for an aldo-keto reductase (ΔdkgB) an alcohol dehydrogenase (ΔyqhD) and the *E. coli* RARE strain [2] (ΔdkgB, ΔyeaE, ΔdkgA, ΔyqhC, ΔyqhD, ΔyjgB, and ΔyahK) were transformed with the Van2 Biosensor system and induced with increasing concentrations of vanillin. The *E. coli* ΔdkgB Van2 (dark blue triangles) and ΔyqhD Van2 (blue diamonds) showed dynamic range of 2.5-fold and 3.17-fold respectively at 2 mM, higher than the *E. coli* BW25113 WT Van2 (pink hexagons) of 2.12-fold. The *E. coli* RARE Van2 (purple circle) showed the highest dynamic range, 4.03-fold at 2mM concentration of vanillin. The fluorescent gene expression normalized to cell density (RFU/OD_600_) is shown. Each value represents the mean and standard deviation of 3 biological replicates.

**Figure S5: A.** SDS-PAGE of purified Van2 and PcaV after TEV cleavage and size exclusion. Both protein bands can be observed in denaturated conditions on the gel with migration on the expected size for the monomer (Van2 16.96 kDa; PcaV 17.06 kDa) and with high purity. **B.** Surface Plasmon Resonance (SPR) sensorgrams of the Van2 and PcaV interaction with the biotinylated DNA palindromic probe PV. Concentrations of Van2 ranging 9.4 nM to 600 nM and PcaV from 0.94 nM to 60 nM were tested.

**
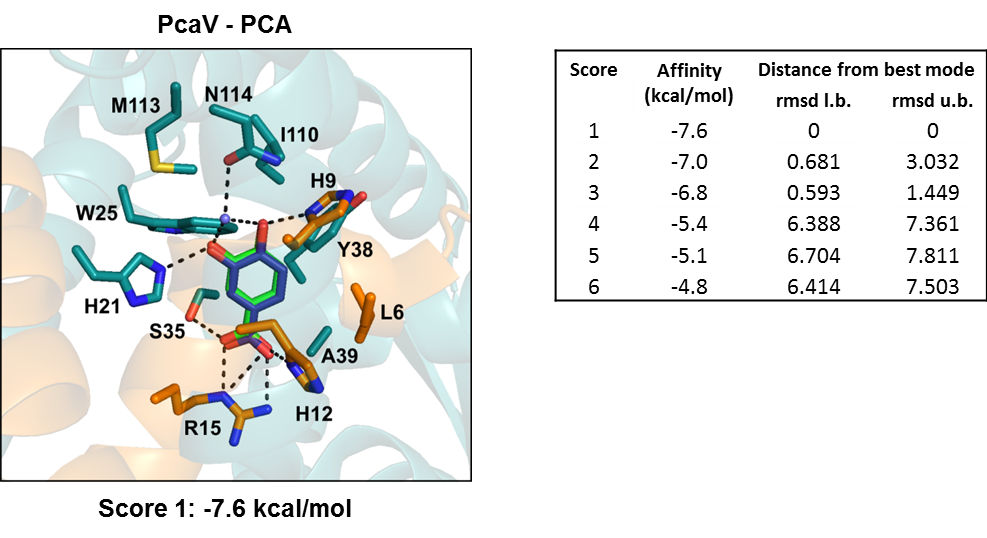
**

**Figure S6:** PcaV re-docking. The protein structure of PcaV complexed to PCA (PDB 4FHT) was used to re-dock PCA using AutoDock Vina [3] to test the docking method. The PCA effector was manually removed from 4FHT and energy was minimized. Re-docking with PCA (green) showed the best score conformation in the same position and similar orientation to the PCA in the crystal structure (dark blue).

**Table S1:** List of oligos.

| **Primer/Probe** | **Sequence 5' - 3'** | **Function** |
| --- | --- | --- |
| **p15 (pcaV DE) FW** | TGAATAAAACGAAAGGCTCAGTCGGAAGACTGG | Amplification of p15 backbone for PcaV DE ligation |
| **p15 (pcaV DE) RV** | ATGCGCTTCCACTTTTTCCCGCGT | Amplification of p15 backbone for PcaV DE ligation |
|  |  |  |
| **van2 pm RV** | AACGCCTAGACGACGATGAACACGCAGACCTTCATCGGTC | PcaV to Van2 Point mutant RV |
| **pcaV pm SA FW** | CGTCTAGGCGTTCGTattGCACGTagtgcgCAGGTGTTTCTGGCA | Point mutant for PcaV M113S N114A |
| **pcaV pm VA FW** | CGTCTAGGCGTTCGTgtgGCACGTatggcgCAGGTGTTTCTGGCA | Point mutant for PcaV I110V N114A |
| **pcaV pm VS FW** | CGTCTAGGCGTTCGTgtgGCACGTagtaacCAGGTGTTTCTGGCA | Point mutant for PcaV I110V M113S |
| **pcaV pm A FW** | CGTCTAGGCGTTCGTattGCACGTatggcgCAGGTGTTTCTGGCA | Point mutant for PcaV N114A |
| **pcaV pm S FW** | CGTCTAGGCGTTCGTattGCACGTagtaacCAGGTGTTTCTGGCA | Point mutant for PcaV M113S |
| **pcaV pm V FW** | CGTCTAGGCGTTCGTgtgGCACGTatgaacCAGGTGTTTCTGGCA | Point mutant for PcaV I110V |
| **van3 FW** | CGTCTAGGCGTTCGTgcgGCACGTagtgcgCAGGTGTTTC | Point mutant for Van3 (V110A) |
| **van4 FW** | CGTCTAGGCGTTCGTctgGCACGTagtgcgCAGGTGTTTC | Point mutant for Van4 (V110L) |
| **van 5 FW** | CGTCTAGGCGTTCGTtggGCACGTagtgcgCAGGTGTTTC | Point mutant for Van5 (V110W) |
| **van6 FW** | CGTCTAGGCGTTCGTgtgGCACGTtgggcgCAGGTGTTTC | Point mutant for Van6 (S113W) |
|  |  |  |
| **PV Probe (IR700 Duplex)** | /5IRD700/TTGACTATACTCAGTGCCCTGACTATGATACT | 5'-InfraRed tagged PV probe for EMSA |
|  | /5IRD700/AGTATCATAGTCAGGGCACTGAGTATAGTCAA |  |
| **Rnd Probe (IR700 Duplex)** | /5IRD700/GAGTTAGAGTACGCGTTAAACACTTCACAGAT | 5'-InfraRed tagged Random probe for EMSA |
|  | /5IRD700/ATCTGTGAAGTGTTTAACGCGTACTCTAACTC |  |
| **PV Probe Bio (5’Biotin Duplex)** | /5Biosg/TTGACTATACTCAGTGCCCTGACTATGATACT | 5'-Biotin tagged PV probe for SPR |
|  | AGTATCATAGTCAGGGCACTGAGTATAGTCAA |  |

**Table S2:** Oligos for PcaV DE library.

| **N.** | **F/R** | **Name** | **Primer sequence** | **Length** |
| --- | --- | --- | --- | --- |
| **1** | F | PcaV DE WT 1 F | GCGGGAAAAAGTGGAAGCGCATATGGCAGCGGTTGATCTGGCAACCCATCCGGGCCACCTGGCCCGCCGT | 70 |
| **2** | F | PcaV DE NNK 1A F | GCGGGAAAAAGTGGAAGCGCATATGGCAGCGGTTGATCTGGCAACCNNKCCGNNKCACCTGGCCCGCCGT | 70 |
| **3** | F | PcaV DE NNK 1B F | GCGGGAAAAAGTGGAAGCGCATATGGCAGCGGTTGATCTGGCAACCNNKCCGGGCCACCTGGCCCGCCGT | 70 |
| **4** | F | PcaV DE NNK 1C F | GCGGGAAAAAGTGGAAGCGCATATGGCAGCGGTTGATCTGGCAACCCATCCGNNKCACCTGGCCCGCCGT | 70 |
| **5** | R | PcaV DE WT 2 R | ACTTGTGGTTTCTTCGCTAACCATGGTATTCCACAGCAGATAGTGTGCCTGCTGCAGACGGCGGGCCAG | 69 |
| **6** | R | PcaV DE MNN 2A R | ACTTGTGGTTTCTTCGCTAACCATGGTATTMNNCAGCAGATAMNNTGCCTGCTGCAGACGGCGGGCCAG | 69 |
| **7** | R | PcaV DE MNN 2B R | ACTTGTGGTTTCTTCGCTAACCATGGTATTMNNCAGCAGATAGTGTGCCTGCTGCAGACGGCGGGCCAG | 69 |
| **8** | R | PcaV DE MNN 2C R | ACTTGTGGTTTCTTCGCTAACCATGGTATTCCACAGCAGATAMNNTGCCTGCTGCAGACGGCGGGCCAG | 69 |
| **9** | F | PcaV DE WT 3 F | TAGCGAAGAAACCACAAGTCCGCAGTATGCTGTTCTGAATGCACTGGTTGCAGAGCCGGGTCTGGATCAG | 70 |
| **10** | F | PcaV DE NNK 3 F | TAGCGAAGAAACCACAAGTCCGCAGNNKGCTGTTCTGAATGCACTGGTTGCAGAGCCGGGTCTGGATCAG | 70 |
| **11** | R | PcaV DE WT 4 R | CTCACCACCTCTGCGATGGTGCTACGATCCAGGCCAACACGTTCACCAACGGTACGCTGATCCAGACCCGG | 71 |
| **12** | F | PcaV DE WT 5 F | CGCAGAGGTGGTGAGCCGTTTGGGCCGTCGGGGTCTGCTGGATAAAGTTCGTGATCCGCAGGATGGTCGTC | 71 |
| **13** | R | PcaV DE WT 6 R | AACGCCTAGACGACGATGAACACGCAGACCTTCATCGGTCAGACGCAGCAGGCTACGACGACCATCCTGCG | 71 |
| **14** | F | PcaV DE WT 7 F | CGTCGTCTAGGCGTTCGTATTGCACGTATGAACCAGGTGTTTCTGGCACCGTTAGCAGCCGATGAACAG | 69 |
| **15** | F | PcaV DE NNK 7A F | CGTCGTCTAGGCGTTCGTNNKGCACGTNNKNNKCAGGTGTTTCTGGCACCGTTAGCAGCCGATGAACAG | 69 |
| **16** | F | PcaV DE NNK 7B F | CGTCGTCTAGGCGTTCGTNNKGCACGTNNKAACCAGGTGTTTCTGGCACCGTTAGCAGCCGATGAACAG | 69 |
| **17** | F | PcaV DE NNK 7C F | CGTCGTCTAGGCGTTCGTNNKGCACGTATGNNKCAGGTGTTTCTGGCACCGTTAGCAGCCGATGAACAG | 69 |
| **18** | F | PcaV DE NNK 7D F | CGTCGTCTAGGCGTTCGTATTGCACGTNNKNNKCAGGTGTTTCTGGCACCGTTAGCAGCCGATGAACAG | 69 |
| **19** | F | PcaV DE NNK 7E F | CGTCGTCTAGGCGTTCGTNNKGCACGTATGAACCAGGTGTTTCTGGCACCGTTAGCAGCCGATGAACAG | 69 |
| **20** | F | PcaV DE NNK 7F F | CGTCGTCTAGGCGTTCGTATTGCACGTNNKAACCAGGTGTTTCTGGCACCGTTAGCAGCCGATGAACAG | 69 |
| **21** | F | PcaV DE NNK 7G F | CGTCGTCTAGGCGTTCGTATTGCACGTATGNNKCAGGTGTTTCTGGCACCGTTAGCAGCCGATGAACAG | 69 |
| **22** | R | PcaV DE WT 8 R | TCTCAGGCCTTCTGCTGCATCTGCAACACGACGAATCAGATCAAAGAAAACGGCCTGTTCATCGGCTGCTAA | 72 |
| **23** | F | PcaV DE WT 9 F | GCAGAAGGCCTGAGAAATCCTGCGGAACCGGCAGTTGCACCGGGTTGAATAAAACGAAAGGCTCAGTCGG | 70 |
| **24** | R | PcaV DE WT 10 R | GTCTTCCGACTGAGCCTTTCG | 21 |

**Table S3:** PcaV DE libraries.

| **Final product** | **Mutated amino acids** | **Primer combination** | **Accuracy (%)** | **Library theoretical size** | **Clones generated** |
| --- | --- | --- | --- | --- | --- |
| **PcaV WT:** | --- | PcaV DE WT 1 to 10. |  |  |  |
| **PcaV DE Library 1 (3aa NNK):** | **I110; M113; N114** | PcaV DE WT 1, 2, 3, 4, 5, 6, 8, 9, 10 and PcaV DE NNK 7A. | 70 % | 3.2 x10^4^ | 2.5 x10^4^ |
| **PcaV DE Library 2 (3aa NNK):** | **H21; Y38; N114** | PcaV DE WT 1, 4, 5, 6 , 8 ,9 ,10; PcaV DE MNN 2C; PcaV DE NNK 3 and PcaV DE NNK 7G. | 62.5 % | 3.2 x10^4^ | 4.0 x10^4^ |
| **PcaV DE Library 3 (3aa NNK):** | **H21; M113; N114** | PcaV DE WT 1, 3, 4, 5, 6, 8, 9, 10; PcaV DE MNN 2C and PcaV DE1 NNK 7D. | 80% | 3.2 x10^4^ | 4.0 x10^4^ |
| **PcaV DE Library 4 (3aa NNK):** | **H9; W25; Y38** | PcaV DE WT 4, 5, 6, 7, 8, 9, 10; PcaV DE NNK 1B; PcaV DE MNN 2B and PcaV DE1 NNK 3. | 62.5 % | 3.2 x10^4^ | 8.0 x10^4^ |

**Table. S4:** List of plasmids.

| **Result section** | **Name** | **Details** | **Antibiotic marker** | **Source** |
| --- | --- | --- | --- | --- |
| **Construction of the PCA Biosensor** | **p44P_LV_eGFP** | p44-pPC-his-eGFP ( phage Lambda based PC reporter of PCA Biosensor) | Ampicillin | This work |
|  | **p44P_PV_eGFP** | p44-pPV-his-eGFP (phage A1 based PV reporter of PCA Biosensor) | Ampicillin | This work |
|  | **p15pcaV** | p15-pLacIpcaV (pcaV transcription factor (original seq. from Streptomyces) | Chloramphenicol | This work |
| **PcaV DE library** | **p15pcaVWT only** | p15-pLacIpcaV (genetic sequence generated with Speedy genes for pcaV transcription factor) | Chloramphenicol | This work |
|  | **p15pcaVDEL1** | p15-pLacIpcaVDE Library1 (pcaV Directed Evolution NNK library of the Amino acids I110,M113 and N114) | Chloramphenicol | This work |
|  | **p15pcaVDEL2** | p15-pLacIpcaVDE Library2 (pcaV Directed Evolution NNK library of the Amino acids H21,Y38 and N114) | Chloramphenicol | This work |
|  | **p15pcaVDEL3** | p15-pLacIpcaVDE Library1 (pcaV Directed Evolution NNK library of the Amino acids H21,M113 and N114) | Chloramphenicol | This work |
|  | **p15pcaVDEL4** | p15-pLacIpcaVDE Library1 (pcaV Directed Evolution NNK library of the Amino acids H9, W25 and Y38) | Chloramphenicol | This work |
| **Biophysical characterisation** | **pET28pcaVWT** | pET28a-pT7_His_TEV_pcaV (His_TEV_PcaV marR aTF expression for biophysical characterisation) | Kanamycin | This work |
|  | **pET28van2** | pET28a-pT7_His_TEV_van2 (His_TEV_Van2 marR aTF expression for biophysical characterisation) | Kanamycin | This work |
| **Point mutational analysis** | **p15van2** | p15 backbone + placI_Van2 (vanillin responsive variant coming from the DE) | Chloramphenicol | This work |
|  | **p15pcaVpmV** | p15 backbone + placI_PcaV I110V (point mutation of PcaV) | Chloramphenicol | This work |
|  | **p15pcaVpmA** | p15 backbone + placI_PcaV N114A (point mutation of PcaV) | Chloramphenicol | This work |
|  | **p15pcaVpmS** | p15 backbone + placI_PcaV M113S (point mutation of PcaV) | Chloramphenicol | This work |
|  | **p15pcaVpmVA** | p15 backbone + placI_PcaV I110V N114A (point mutation of PcaV) | Chloramphenicol | This work |
|  | **p15pcaVpmVS** | p15 backbone + placI_PcaV I110V M113S (point mutation of PcaV) | Chloramphenicol | This work |
|  | **p15pcaVpmSA** | p15 backbone + placI_PcaV M113S N114A(point mutation of PcaV) | Chloramphenicol | This work |
|  | **p15van3** | p15 backbone + placI_van3 (V110A) (point mutation of van2) | Chloramphenicol | This work |
|  | **p15van4** | p15 backbone + placI_van4 (V110L) (point mutation of van2) | Chloramphenicol | This work |
|  | **p15van5** | p15 backbone + placI_van5 (V110W) (point mutation of van2) | Chloramphenicol | This work |
|  | **p15van6** | p15 backbone + placI_van6 (S113W) (point mutation of van2) | Chloramphenicol | This work |

**Table. S5:** List of strains.

| **Name** | **Details** | **Source** |
| --- | --- | --- |
| ***E. coli* DH 5-alpha** | Cloning strain [F^–^ *endA1* *glnV44* *thi-1* *recA1* *relA1* *gyrA96* *deoR* *nupG* *purB20* φ80d*lacZ*ΔM15 Δ(*lacZYA-argF*)U169, hsdR17(*r_K_*^–^*m_K_*^+^), λ^–^ ] | *New England Biolabs* |
| ***E. coli* Bl21** | Expression strain [F^–^ *ompT* *gal* *dcm* *lon* *hsdS_B_*(*r_B_*^–^*m_B_*^–^) [*malB*^+^]_K-12_(λ^S^) *araB*::*T7RNAP-tetA*] | *New England Biolabs* |
| ***E. coli* BW25113** | *Parent strain of the Keio collection* [*lacI*^+^*rrnB*_T14_ Δ*lacZ*_WJ16_ *hsdR*514 Δ*araBAD*_AH33_ Δ*rhaBAD*_LD78_ *rph-1Δ(araB–D)567 Δ(rhaD–B)568 ΔlacZ4787(::rrnB-3) hsdR514 rph-1*] | Keio collection (NIG-Japan) |
| ***E. coli* BW25113 ΔdkgA** | *E. coli* BW25113 Knockout [*ΔdkgA*] | Keio collection (NIG-Japan) |
| ***E. coli* BW25113 ΔdkgB** | *E. coli* BW25113 Knockout [*ΔdkgA*] | Keio collection (NIG-Japan) |
| ***E. coli* BW25113 ΔyeaE** | *E. coli* BW25113 Knockout [*ΔyeaE*] | Keio collection (NIG-Japan) |
| ***E. coli* BW25113 ΔyqhD** | *E. coli* BW25113 Knockout [*ΔyqhD*] | Keio collection (NIG-Japan) |
| ***E. coli* BW25113 ΔyahK** | *E. coli* BW25113 Knockout [*ΔyahK*] | Keio collection (NIG-Japan) |
| ***E. coli* BW25113 ΔyjgB** | *E. coli* BW25113 Knockout [*ΔyjgB*] | Keio collection (NIG-Japan) |
| ***E. coli* RARE** | *E. coli* K-12 MG1655 Knockout [*ΔdkgB, ΔyeaE, ΔdkgA, ΔyqhC, ΔyqhD, ΔyjgB, and ΔyahK*] | Kunjapur *et al*, 2014 (Addgene) |

**Table S6:** Chemicals for PCA substrates screening.

| **Name** | **CAS** | **Supplier** |
| --- | --- | --- |
| **trans-Ferulic acid** | 537-98-4 | Sigma |
| **p-Coumaric acid** | 501-98-4 | Sigma |
| **Sinapic acid** | 530-59-6 | Sigma |
| **Gallic acid** | 149-91-7 | Sigma |
| **Syringic acid** | 530-57-4 | Sigma |
| **Protocatechuic acid (3,4-Dihydroxybenzoic acid)** | 99-50-3 | Sigma |
| **Vanillic acid** | 121-34-6 | Sigma |
| **4-Hydroxy-3-methylbenzoic acid** | 499-76-3 | Sigma |
| **Terephthalic acid** | 100-21-0 | Sigma |
| **2,5-Furandicarboxylic acid** | 3238-40-2 | Sigma |
| **2,5-Thiophenedicarboxylic acid** | 4282-31-9 | Sigma |
| **Furoic acid** | 88-14-2 | Sigma |
| **trans,trans-Muconic acid** | 3588-17-8 | Sigma |
| **cis,cis-Muconic acid** | 1119-72-8 | Sigma |
| **Benzoic acid** | 65-85-0 | Sigma |
| **4-Hydroxybenzoic acid** | 99-96-7 | Sigma |
| **Caffeic acid** | 331-39-5 | Sigma |
| **Vanillin** | 121-33-5 | Sigma |
| **3-Hydroxybenzoic acid** | 99-06-9 | Sigma |
| **2-Hydroxybenzoic acid (Salicylic acid)** | 69-72-7 | Sigma |
| **3,5-Dihydroxybenzoic acid** | 99-10-5 | Sigma |
| **2,4-Dihydroxybenzoic acid** | 89-86-1 | Sigma |
| **2,5-Dihydroxybenzoic acid** | 490-79-9 | Sigma |
| **Methyl 3,4-dihydroxybenzoate** | 2150-43-8 | Fluorochem |
| **3,4-dihydroxybenzamide** | 54337-90-5 | Fluorochem |
| **p-phenylenediamine** | 106-50-3 | Sigma |
| **Phthalic acid** | 88-99-3 | Sigma |
| **4-Vinylphenol** | 2628-17-3 | Sigma |
| **Methyl benzoate** | 93-58-3 | Sigma |

**Table S7:** Chemical targets for PcaV Directed Evolution.

| **Name** | **CAS** | **Supplier** |
| --- | --- | --- |
| **Vanillin** | 121-33-5 | Sigma |
| **Terephthalic acid** | 100-21-0 | Sigma |
| **2,5-Furandicarboxylic acid** | 3238-40-2 | Sigma |
| **Caffeic acid** | 331-39-5 | Sigma |
| **trans-Ferulic acid** | 537-98-4 | Sigma |
| **3,4-Dihydroxybenzoic acid (Protocatechuic acid)** | 99-50-3 | Sigma |

**Table S8:** Chemicals for Van2 substrates screening.

| **Name** | **CAS** | **Supplier** |
| --- | --- | --- |
| **3,4-Dihydroxybenzoic acid (Protocatechuic acid)** | 99-50-3 | Sigma |
| **Vanillin** | 121-33-5 | Sigma |
| **Vanillic acid** | 121-34-6 | Sigma |
| **4-Hydroxy-3-methoxybenzyl alcohol** | 498-00-0 | Sigma |
| **4-Hydroxybenzaldehyde** | 123-08-0 | Sigma |
| **4-Hydroxy-3-methoxycinnamaldehyde (coniferyl aldehyde)** | 458-36-6 | Sigma |
| **3-Hydroxy-4-methoxybenzaldehyde** | 621-59-0 | Sigma |
| **3,4-Dimethoxybenzaldehyde** | 120-14-9 | Sigma |
| **4-Benzyloxy-3-methoxybenzaldehyde** | 2426-87-1 | Sigma |
| **Guaiacol** | 90-05-1 | Sigma |
| **Benzaldehyde** | 100-52-7 | Sigma |
| **2-Nitrobenzaldehyde** | 552-89-6 | Alfa Aesar |
| **Phenylacetaldehyde** | 122-78-1 | Sigma |
| **Indole-3-carboxaldehyde** | 487-89-8 | Sigma |
| **5-Hydroxymethylfurfural** | 67-47-0 | Sigma |
| **Furfural** | 98-01-1 | Sigma |
| **4-nitrobenzaldehyde** | 555-16-8 | Alfa Aesar |
| **3-Methoxybenzaldehyde** | 591-31-1 | Alfa Aesar |
| **3,4-Dihydroxybenzaldehyde** | 139-85-5 | Sigma |
| **4-Hydroxy-3-nitrobenzaldehyde** | 3011-34-5 | Sigma |
| **Syringaldehyde** | 134-96-3 | Sigma |
| **2-Hydroxybenzaldehyde** | 90-02-8 | Sigma |
| **2-Methoxybenzaldehyde** | 135-02-4 | Sigma |
| **2-Hydroxy-3-methoxybenzaldehyde** | 148-53-8 | Sigma |
| **2,4-Dihydroxybenzaldehyde** | 95-01-2 | Sigma |
| **2,3-Dimethoxybenzaldehyde** | 86-51-1 | Sigma |
| **3,5-Dimethoxybenzaldehyde** | 7311-34-4 | Sigma |
| **2-Hydroxy-5-methoxybenzaldehyde** | 672-13-9 | Sigma |
| **2,3-Dihydroxybenzaldehyde** | 24677-78-9 | Sigma |
| **4-Hydroxy-2-methoxybenzaldehyde** | 18278-34-7 | Sigma |
| **3,4-Dihydroxy-5-methoxybenzaldehyde** | 3934-87-0 | Sigma |
| **4-Hydroxy-3-methylbenzaldehyde** | 15174-69-3 | Sigma |

**Table S9:** Docking scores AutoDock Vina.

|  | **Score** | **Affinity (kcal/mol)** | **Distance from best mode** | |
| --- | --- | --- | --- | --- |
|  |  |  | **rmsd l.b.** | **rmsd u.b.** |
| **Van2-vanillin** | 1 | -6.4 | 0 | 0 |
|  | **2** | **-6.1** | **2.671** | **5.329** |
|  | 3 | -5.9 | 2.642 | 4.44 |
|  | 4 | -5.8 | 1.445 | 3.548 |
|  | 5 | -5.1 | 7.953 | 9.014 |
|  | 6 | -4.7 | 6.562 | 7.415 |
|  | 7 | -4.3 | 1.251 | 2.202 |
|  | 8 | -4.3 | 6.143 | 9.084 |
|  | 9 | -4.2 | 6.145 | 10.014 |
|  | 10 | -4.1 | 6.154 | 9.122 |
|  |  |  |  |  |
| **PcaV M113S N114A - vanillin** | 1 | -6.4 | 0 | 0 |
|  | **2** | **-6.1** | **2.564** | **5.021** |
|  | 3 | -6 | 2.408 | 4.236 |
|  | 4 | -5.7 | 1.36 | 3.392 |
|  | 5 | -4.2 | 2.224 | 4.02 |
|  | 6 | -4.1 | 1.486 | 2.307 |
|  | 7 | -4.1 | 5.918 | 8.835 |
|  | 8 | -4.1 | 5.925 | 9.694 |
|  | 9 | -4.1 | 5.892 | 8.832 |
|  | 10 | -3.9 | 3.52 | 5.098 |
|  |  |  |  |  |
| **PcaV - PCA** | **1** | **-7.6** | **0** | **0** |
|  | 2 | -7.0 | 0.681 | 3.032 |
|  | 3 | -6.8 | 0.593 | 1.449 |
|  | 4 | -5.4 | 6.388 | 7.361 |
|  | 5 | -5.1 | 6.704 | 7.811 |
|  | 6 | -4.8 | 6.414 | 7.503 |

**Table S10.** Dose response curve fitting of the PCA Biosensor.

| **PCA biosensor promoter** | **Min signal** | **Max signal** | **Max/Min** | **Hill coefficient** | **EC50**  **(µM)** |
| --- | --- | --- | --- | --- | --- |
| P_PV_ Biosensor | 106.4 ± 210.9 | 16076.4± 520.8 | 151.1 ± 17.1 | 1.6 ± 0.1 | 364.3 ± 20.1 |
| P_LV_ Biosensor | 109.5 ± 122.7 | 10288.1 ± 802.1 | 93.9 ± 11.9 | 1.3± 0.1 | 837.4 ± 111 |
| **PCA biosensor specificity** |  |  |  |  |  |
| PCA | 88.0 ± 6.3 | 16852.0 ± 705.4 | 191.5±15.2 | 1.4 ± 0.1 | 379.3 ± 33.4 |
| 3HB | 78.4 ± 10.0 | 15027.4 ± 55.8 | 191.7 ± 3.4 | 2.5 ± 0.1 | 89.2 ± 1.6 |
| 4HB | 82.9 ± 4.4 | 10762.6 ± 52.7 | 129.8 ± 2.4 | 2.6 ± 0.1 | 443.3 ± 3.5 |

**Bibliography:**

1. Davis JR, Brown BL, Page R, Sello JK. Study of PcaV from Streptomyces coelicolor yields new insights into ligand-responsive MarR family transcription factors. Nucleic Acids Res. 2013;41:3888–900. doi:10.1093/nar/gkt009.

2. Kunjapur AM, Tarasova Y, Prather KLJ. Synthesis and Accumulation of Aromatic Aldehydes in an Engineered Strain of Escherichia coli. J Am Chem Soc. 2014;136:11644–54. doi:10.1021/ja506664a.

3. Trott O, Olson AJ. AutoDock Vina: Improving the speed and accuracy of docking with a new scoring function, efficient optimization, and multithreading. J Comput Chem. 2009;31:NA-NA. doi:10.1002/jcc.21334.
